# The Effect of Teenage Passengers on Simulated Risky Driving Among Teenagers: A Randomized Trial

**DOI:** 10.3389/fpsyg.2019.00923

**Published:** 2019-04-30

**Authors:** Bruce G. Simons-Morton, C. Raymond Bingham, Kaigang Li, Chunming Zhu, Lisa Buckley, Emily B. Falk, Jean Thatcher Shope

**Affiliations:** ^1^Eunice Kennedy Shriver National Institute of Child Health and Human Development, Bethesda, MD, United States; ^2^Transportation Research Institute, University of Michigan, Ann Arbor, MI, United States; ^3^Health and Exercise Science, College of Health and Human Sciences, Colorado State University, Fort Collins, CO, United States; ^4^The Professional Group, Glotech Team, Bethesda, MD, United States; ^5^Transport and Road Safety Research, School of Aviation, University of New South Wales, Sydney, NSW, Australia; ^6^Annenberg School for Communication, Wharton Marketing Department, and Department of Psychology, University of Pennsylvania, Philadelphia, PA, United States

**Keywords:** risk behavior, driving simulator, Cyberball, conformity, social exclusion, social norms

## Abstract

Teenage passengers might influence risky driving, particularly in certain mental states. Notably, social exclusion could increase social conformity. Two studies examined simulated intersection management among young drivers after a social exclusion activity (Cyberball). In Study 1 [112 males (mean = 17.3 years)], risky driving was significantly greater among excluded males driving with a risk-accepting vs. passive passenger; no effect of social exclusion. In Study 2 [115 females (mean = 17.1 years)], risky driving was significantly greater among excluded females driving with a risk-accepting vs. a passive passenger, and greater among those included (fair play) vs. excluded when driving with a risk-accepting passenger. Risky driving behavior among male and female teenagers may be influenced uniquely by passenger norms and social exclusion.

## Introduction

High crash rates among novice teenage drivers are thought to be due to deficiencies in driving skill and judgment due to young age (Twisk and Stacey, 2007), inexperience; (McKnight and McKnight, 2003; Simons-Morton et al., 2011), and risky driving behavior (Williams, 2003; Curry et al., 2011; Simons-Morton et al., 2011, 2015; Peake et al., 2013). Risky driving among teenagers is thought to vary according to driving conditions, including passenger presence (Ouimet et al., 2015; Simons-Morton and Ouimet, 2017). Moreover, the influence of teenage passenger presence may vary according to the mental state of the teenage driver (Falk et al., 2014).

Fatal crash risk is lower with adult passengers, but higher with teenage passengers, particularly among teenage drivers (Ouimet et al., 2010). A recent systematic review found relatively consistent evidence for an association between passenger presence and fatal crash outcomes, with odds ratios ranging from 1.24 to 1.89 across studies, increasing to 1.70–2.92 for two or more passengers, and with higher risk among male than female drivers and younger versus older young drivers (Ouimet et al., 2015). Fatal crashes tend also to involve high speeds, inclement weather, and late-night driving, so passenger presence is only one important factor. Curiously, teenage passenger presence was inconsistently associated with crash risk in studies that examined non-fatal or the combination of fatal and non-fatal crashes (Ouimet et al., 2015), which are vastly more prevalent, if less harmful, than fatal crashes. A tentative conclusion of the systematic review was that crash risk in the presence of teen passengers might be higher or lower depending on characteristics of the driver and the passenger.

Passenger influences on teenage driver behavior are thought to occur through social influence and/or distraction (Ouimet et al., 2015; Simons-Morton and Ouimet, 2017). Teenage passenger influences may be conditional, with some teenage passengers increasing risk among some teenage drivers under certain conditions and decreasing risk under other conditions (Ouimet et al., 2015; Simons-Morton et al., 2016). Notably, risky driving behaviors are greater when the driver perceives that peer norms favor these behaviors (Simons-Morton et al., 2011), when the driver is sensitive to social threats (Falk et al., 2014), and when the driver is emotionally aroused (Abdu et al., 2012; Taubman-Ben-Ari, 2012). Hence, it is of interest to examine passenger influences on risky driving behavior in variable driver mental states.

Simulation, even in an actual vehicle with high fidelity sounds and motion representing acceleration, braking, and turning; and realistic graphics of scenarios based on actual roads, cannot fully capture actual on-road driving experience. However, simulated driving performance has consistent been associated with on-road performance (Mullen et al., 2011) and has the decided advantage of being completely safe. Therefore, simulation can be a useful method for experimentation, allowing experimental manipulation that could not be done safely in traffic. Three recent randomized trials reported significant effects of passenger presence on the simulated risky-driving behavior of young drivers. In these studies, simulated risky driving was measured variably, but each assessed failure to react to a stop signal or stop at red lights positioned carefully within the scenarios and timed to require the driver to make immediate decisions to stop or risk running some of the lights. Ross et al. (2016) compared the simulated risky driving in the presence of each participant’s own peer as the passenger in samples of 17–18 (*n* = 30) and 21–24 (*n* = 20) year-old males and females. Key measures of risky driving included average speed and reaction time to a stop signal at variable time intervals. Among drivers in both age groups, red light running was greater in the presence of passengers. Also, among participants with low inhibitory control, speeding was more prevalent in the presence of passengers. However, passenger presence seemed to improve hazard management and reduced time in the intersection when the light was red, providing additional support for the contention that peer passengers can increase some risks and decrease others, possibly conditional on characteristics of the driver, passenger, and/or driving conditions.

Bingham et al. (2016) examined the effect of norms and peer pressure on red light management and the decision to pass a slowing lead vehicle. Licensed male teenagers (*n* = 53) were randomized to drive with a young male passenger (a study confederate) who in the risk-promoting group presented himself as risk accepting and when riding as the passenger exercised mild peer pressure to complete the course quickly; those in the other group drove with the confederate passenger who presented himself as risk averse and when riding as the passenger exercised mild pressure to complete the drive safely by taking few risks. Risky driving (running a red light, time in the intersection, and passing the slowing vehicle) and distraction (failure to stop at an intersection with an occluded stop sign) were greater in the passenger compared to the solo drives, a main effect for passenger presence, consistent with theory and research indicating that adolescent reward sensitivity increases in the presence of peers (Chein et al., 2011). In addition, Bingham et al. (2016) found interactions by passenger type where, relative to the group that experienced mild passenger pressure-to-drive safely, those who experienced mild pressure-to-take-risks ran more red lights and were more likely to pass the slowing vehicle. These findings are consistent with the contention that risk in the presence of passengers is conditional on peer pressure.

Simons-Morton et al. (2014) examined the effect of social norms without overt pressure on simulated risky driving measures identical to Bingham et al. (2016). Young male drivers (*n* = 66) were randomized to drive solo and with a confederate passenger portraying either risk-accepting or risk-averse social norms. The results confirmed the independent effect of passenger presence and significant interactions by passenger social norms, with those in the group exposed to the confederate passenger with risk-accepting norms, relative to those exposed to the passenger with risk-averse social norms, more likely to run the red light and spend more time in the intersection while the light was red. These findings are consistent with other research indicating that teenage risk taking is greater in the presence of peers, perhaps by sensitizing the brain’s reward system to risk taking (Chein et al., 2011), conditional on passenger social norms (Ouimet et al., 2015).

### Social Exclusion and Risky Driving

In the study just described (Simons-Morton et al., 2014), a week before driving the simulator, in an fMRI setting in which participants’ brain activity was assessed, participants played the Cyberball (social exclusion) game (Falk et al., 2014). Cyberball is a computerized game of “catch” in which the participant (using a mouse) and other (unseen) players pass a “ball” on the computer screen visible in the scanner (Williams and Jarvis, 2006). Although the participant is made to believe he is playing with two other actual people, a pre-set computer program, rather than the other players, controls the ball’s movement from other players. Thus, initially all players receive the ball approximately equally (i.e., fair play). In a later “exclusion” round, however, the other participants (the computer actually) stop passing the ball to the participant. When excluded, participants experience variable levels of distress or social pain. Eisenberger argues that the neural basis of rejection is that the pain system has co-opted the social attachment system, making social rejection among the most “painful” human experiences. Accordingly, individual differences in increased activity in neural systems associated with distress during the exclusion task predicted increased simulated risky driving the following week in the presence of a confederate peer passenger (Falk et al., 2014).

Likewise, in the study by Bingham et al. (2016), participants also played Cyberball in an fMRI scanner one week before the driving simulator experiment. In that study, the extent to which participants’ brains changed their patterns of connectivity between the inclusion (fair play) and exclusion conditions predicted the degree to which they later conformed to the passenger norms in the subsequent driving simulator session (Wasylyshyn et al., 2018). Both sets of findings are consistent with literature demonstrating that greater sensitivity to exclusion is associated with conformity to peer norms (Williams and Nida, 2011; Falk et al., 2012). Other studies have shown that this is particularly true among those low in resistance to peer influence (Steinberg and Monahan, 2007; Peake et al., 2013). Thus, sensitivity to social pain, and more broadly social cues, such as cues experienced when excluded during Cyberball and measured by social pain and mentalizing regions in the brain during the task (Falk et al., 2014; Wasylyshyn et al., 2018), is thought to increase subsequent conformity to normative behavior as a means of social compensation (Williams and Nida, 2011). According to the need-threat model of ostracism, we expected that experiencing social rejection prior to driving in the presence of a peer would threaten psychological needs such as self-esteem, control, and belonging (Bastian and Haslam, 2010; Pharo et al., 2011; Williams and Nida, 2011). In this case, social exclusion could lead to subsequent conformity to peers’ risk-taking preferences to attain or regain acceptance and avoid rejection (DeWall, 2010; Spear, 2011; Williams and Nida, 2011; Falk et al., 2012). On the other hand, prior work notes that social exclusion prompts attempts at re-connection (e.g., through conformity) only when people expect to be able to easily connect with subsequent interaction partners (Maner et al., 2007). It is also possible that the boost in reward sensitivity and risk behavior, observed in the presence of peers in prior studies (Chein et al., 2011), would be augmented in the presence of a study confederate who is liked and/or when the participant believes there is a high likelihood of connection. In this case, if a recent experience of being socially included or at least treated fairly signals a greater possibility of later social inclusion, conformity to a risk promoting peer could be higher following inclusion (fair play) than exclusion.

### Study Purpose

To examine the conditional effects of teenage passengers on risky driving, we conducted two randomized trials in which we measured simulated driving behavior among teenagers in the presence of confederate peer passengers immediately after drivers were either socially excluded or included during a computer activity. The current research builds on the findings of previous driving studies of passenger effects on risky driving and on the finding just described that individual variability in the brain’s sensitivity to exclusion was associated with greater susceptibility to peer influence on teenage male risky driving one week later (Falk et al., 2014; Wasylyshyn et al., 2018). The purpose of the current research is to evaluate the effect of experimental manipulation of social exclusion vs. inclusion (fair play) on male and female teenage simulated risky driving in the presence of confederate peer passengers who exhibited either risk accepting or passive social norms with respect to risky driving. Two research questions were examined: (a) What is the effect on simulated risky driving of exposure to a risk accepting or passive passenger after social exclusion? (b) What is the effect on simulated risky driving of social exclusion or social inclusion (fair play) when exposed to a risk accepting passenger?

## Materials and Methods

### Design

Two simulation studies were conducted using the same methods and procedures. Study 1 included 112 males 15 to 18 years old (mean = 17.3) and Study 2 included 115 females 16 to 18 years old (mean = 17.1); participants had a Level 2 Michigan driver license (allowing independent driving with restrictions). Participant assent and parent consent were obtained and participants were compensated according to the protocol approved by the University of Michigan IRB.

**FIGURE 1:**
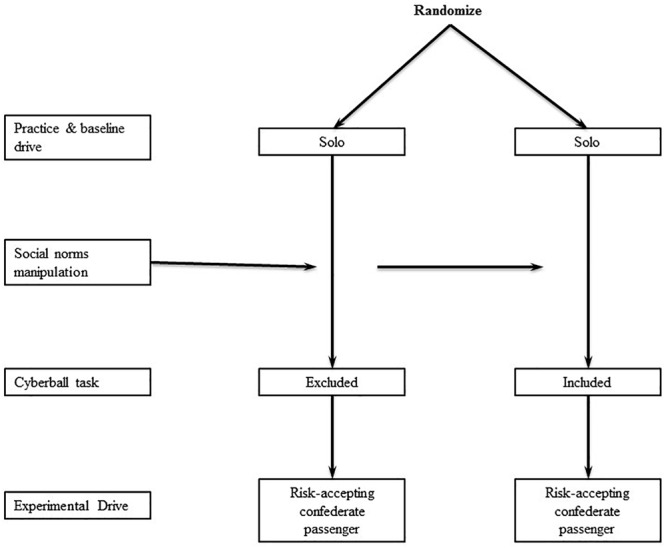
Team passenger study design.

In the between subject designs (shown in Figure 1), participants were randomly assigned to drive solo and in one of three groups with a male confederate passenger: (a) exclusion with passive passenger presence (Exclusion + Passive passenger); (b) exclusion with risk accepting passenger presence and norms (Exclusion + Risk accepting passenger); and (c) inclusion (i.e., fair play condition) with risk accepting passenger presence and norms (Inclusion + Risk accepting passenger). (A full factorial design was not feasible within the available resources.) Exclusion was manipulated by computer activity as programmed in the Cyberball computer activity. Those assigned to drive with a risk-accepting passenger were exposed to a social-norms priming activity. Participants in the Exclusion + Passive passenger group were not exposed to the social norms priming activity, but drove with a passive (i.e., not risk-accepting) confederate passenger who interacted minimally with the participant.

### Social-Norms Priming Manipulation

Consistent with prior research on peer driving norms (Simons-Morton et al., 2014; Bingham et al., 2016), predrive social norms-priming activities, conducted with those assigned to the risk accepting passenger groups, included two confederate passenger activities: (a) arriving late, explaining (“Sorry I was a little late getting here. Normally I drive way faster, but I hit like every red light.”); and (b) watching and rating with the participant two driving videos, the first providing a view from the passenger seat of being in a car racing at high speed, weaving in and out of traffic on an expressway, the second of being in a car driven carefully at a slower speed than the other expressway vehicles. Immediately after each video the participant was asked to respond verbally to two questions on a scale of 1 to 10: (a) How similar is your driving to the driver in the video? and (b) How likely would you be to ride with the person in this video? The confederate responded after the participant so that he could always respond in a manner that was more-or-less risk accepting, depending on treatment condition, relative to the participant’s response. The experimenter then indicated that the study participant had been randomly selected to be the driver for the experiment and the confederate was assigned to be the passenger. The research assistant then announced that the passenger (confederate) would drive the simulated vehicle while the participant played the Cyberball game in another room.

### Manipulation of Social Exclusion or Inclusion (Fair Play) Using Cyberball Approach

Cyberball has been validated as a reliable way of simulating the experience of social exclusion (Williams, 2007; Eisenberger, 2012). The experimenter explained that Cyberball was (“a virtual ball tossing game and participants will be playing the game live with two other participants who are in other rooms”). The participant was then logged on to a virtual room in which he or she encountered two additional players (controlled by a preset computer algorithm). Participants thought they were playing teens other than the confederate, but the game was controlled by a computer program. A fair game of Cyberball was always played first, in which the participant and two virtual players received the ball equally often. For those in the exclusion condition, this fair game was followed by an unfair game, in which the participant and virtual players started out receiving the ball equally often, but after the first few throws, the other players stopped throwing to the participant all together, simulating social exclusion. Those randomized to inclusion (fair play) experienced fair play through out and received the ball equally as often as the other players. At the completion of the Cyberball game, which took 6 to 7 min to play, the participants completed a survey that assessed their reactions to the game.

### Equipment

A fixed-base high-fidelity simulator located in a dedicated lab space was used for this study. The simulator comprised a full vehicle cab (Nissan Versa) surrounded by three forward screens and one rear screen. The forward screens were projected at a resolution of 1400 × 1050 pixels each and the rear screen at 1024 × 768 pixels, providing a 120-degree forward field of view and a 40-degree rear field of view. The simulator runs RTI’s (Realtime Technologies, Inc., Royal Oak, MI, United States) SimCreator software. The simulator system included steering feedback, road vibration, a virtual LED instrument cluster, sideview mirrors, and simulated audio. The driving simulator recorded vehicle and driving performance data, up to six synchronized channels of video, and two channels of audio at 30 Hz (see Appendix 1).

### Procedure

During the experimental drives, the confederate (as either risk accepting or passive) was passive with respect to risk and rode quietly to minimize variability in passenger behavior. Participants completed three drives: 5-min coaching/practice drive; 10-min baseline (solo) drive (after which participants played the Cyberball game); and 15-min experimental (passenger) drive. All three drives included typical roadway features (e.g., four-way intersections, straight and curved rural road, expressway) and a wide range of roadway geometries, speed limits, traffic conditions, and visual elements. The drives differed in the ordering of residential, rural, urban, and freeway road segments, with distinct layouts and alterations to surface features (e.g., trees, buildings), but included identical driving scenarios for eliciting participant behavior, including a car passing task and multiple four-way signalized intersections. Construction barrels at intersections and junctions guided participants to the destination and included a lead vehicle that served to minimize variability in the speeds at which intersections were encountered.

Intersection management, particularly when in the dilemma zone when the light turns amber as the driver approaches the intersection and must quickly decide to brake sharply or pass through the intersection as or after the light turns red, which is a traffic violation and dangerous behavior zone’ (Huang et al., 2008). Accordingly, participants encountered signalized intersections at periodic intervals of 13 to 15 s (at 35 mph), exposing them to green and yellow lights of different durations (2.6, 3.0, 3.4 s), and red lights. The different durations of lights sometimes forced participants to choose to stop without entering the intersection, go through the intersection before the light turned red, or be caught in a ‘dilemma.’ The measurement of signalized intersection management is useful for several reasons: intersection are a common driving experience; intersection crashes are relatively common and often result in serious damage; there is considerable variability within and between drivers in intersection management; and it is possible to introduce in a single drive multiple intersections, including many that place drivers in the “dilemma zone,” where the light turns yellow and the driver must make a quick decision to stop or go (Liu and Herman, 1996).

### Measures

#### Outcomes

The average treatment group percent was calculated for three intersection management/risky driving measures: (a) stopping for the red light (% Failed to Stop) in the 10 (of 18) intersections with relatively shorter durations between yellow lights (i.e., dilemma zone intersections); this measured the percent of appropriate stopping at the 10 short duration lights; (b) time vehicles were in intersections while the light was red light (% Time in Red); the measure assessed the average amount of time the was in the intersection while the light was red as a reflection of the duration of potential risk for a crash; and (c) passing the slowing lead vehicle (% Passed Slow Vehicle); passing the slowing vehicle represented greater acceptance of risk.

#### Baseline Tests of Randomization

To assess individual variability, the week prior to the exclusion task and simulation drives participants completed the following baseline measures: susceptibility to peer pressure (Steinberg and Monahan, 2007), included 10 items that asked “what would you do if…,” with response options of no (1), probably not (2), probably (3), and yes (4); self-esteem (Robins et al., 2001) is a single item, “I have high self-esteem,” with response options from strongly disagree (1) to strongly agree (5); risky driving behavior (Simons-Morton et al., 2015) includes 28 items with response options from never (1) to always (5); and perceived social status (Adler and Stewart, 2007) is a single item that asks respondents to rate themselves from the bottom (1) to the top (1) of the ladder of people in the United States who are best off; and demographics; and impulsive behavior was assessed with 16 items from the UPPS (Cyders et al., 2007) with response options from strongly disagree (1) to strongly agree.

#### Effect of Social Exclusion

Cyberball has been validated in behavioral and neuroimaging studies to simulate the experience of social exclusion (Williams, 2007; Eisenberger, 2012). Immediately after playing the Cyberball game participants were asked, “How much did they throw you the ball?” with options from 1 = not at all to 5 = a lot. Then participants completed the 20 item Need-Threat Scale (van Beest and Williams, 2006; Williams, 2007) that asked their agreement (1 = strongly disagree to 7 = strongly agree) to five questions in each of the following four subscales: belonging (e.g., “I felt as one with the other players”); self-esteem [e.g., “Playing the game made me insecure (reverse coded)”]; meaningful existence (e.g., “I felt in control over the game”); and control (e.g., I think my participation in the game was useful”). Higher scores reflect lower psychological need.

#### Effect of Social Norms Priming

The following items, administered in a post-drive survey, were adapted or created for this study and provide additional information about the participants’ experience. *Identification with passenger* was measured by six items that asked participants to indicate (1 = no, 2 = maybe, 3 = yes) their identification with the passenger (i.e., Is the passenger someone you would like to know better or someone you liked?). *Passenger approval*, a measure of subjective norms, was measured by five items asking participants how likely it was (1 = very unlikely to 5 = very likely) that the passenger would approve of the participant’s involvement in five risky driving behaviors such as driving 10 mph above the speed limit and closely following a slow vehicle.

### Power and Sample Size

Power analysis was based on data from previous simulation studies (Simons-Morton et al., 2014; Bingham et al., 2016) for the variable, percentage of correct stops at 18 yellow light intersections that invoke a stopping dilemma. Accordingly, an effect size of 0.53 was expected. Thus, detecting a treatment group difference of this magnitude with a power of 0.80 and alpha of 0.05 a sample size of 40 per group is required. Given the experimental design and counterbalancing requirements, the three-group design requires a total sample size of 120 participants for each study.

### Statistical Analysis

Treatment group differences on the pre-drive randomization and post-drive assessment variables (evaluating passenger norms manipulation) were assessed using one-way ANOVA (2X2 ANOVA might bias against possible effects) and *post hoc* comparisons with Tukey–Kramer adjustment. Between treatment groups psychological needs differences for excluded and included participants were assessed after the exclusion task using independent *t*-tests.

The primary driving performance comparisons were examined as the differences (passenger minus solo drive) of the treatment groups on each measure of risky driving. The solo drive served to control for individual differences in driving behavior. PROC GLIMMIX in SAS (version 9.4) was used to fit generalized linear mixed models (GLMMs) where the outcomes were % Not Stopping for Red light (average of binary outcomes generating odds ratios), % Time in Red (normal outcome of the average across multiple intersections generating β, and % Passing Slowing Vehicle (average of binary outcomes generating odds ratios). The GLIMMIX model follows:

$$\begin{array}{c} \mu =\beta_{0}+\beta_{1}\;(BaseExp)(Condition1)+\beta_{2}(BaseExp)(Condition2)+\beta_{3}(BaseExp)(Condition3)+b_{i} \end{array}$$

where $\mu =log(\frac{\pi }{1-\pi })$ for the variables pf “Failure to Stop at a red light” (binary variable), “Pass Slow Vehicle” (binomial random variable) and “mean % Time in Red” (continuous variable); BaseExp (0 = solo baseline and 1 = experimental driving with confederate); *b_i_* denotes a subject specific random effect, β_0_ denotes baseline value, and *β*_1_, *β*_2_, and *β*_3_ characterize the effect of each exclusion/inclusion and passenger risk comparison. There were two treatment group comparisons (in relation to baseline values): comparison 1 was the effect of a risk accepting vs. passive passenger given exclusion, where 1 = Excluded + Passive passenger; 2 = Excluded + Risk accepting passenger; Comparison 2 was the effect of inclusion (fair play) vs. exclusion, given a risky passenger; 1 = Exclusion + Risk accepting and 2 = Inclusion (fair play) + Risk accepting Passenger. The models were then rerun adjusting separately for baseline self-esteem and susceptibility to peer pressure. Odds ratios are considered the effect size for GLIMMIX models with binary outcomes and the beta is the effect size for mixed models. In addition, we calculated the standardized mean treatment group differences.

## Results

### Study Participants

In Study 1, 112 of the 134 recruited participants and in Study 2, 115 of the 137 recruited participants completed the protocol and were included in the analyses. Exclusions were due to simulator sickness or technical issues with the simulator, a rate that is consistent with other driving simulator studies (Caird and Horrey, 2011). As a check on randomization we assessed at baseline, before any treatment group manipulation or simulated driving, measures of self-esteem, susceptibility to peer pressure, risky driving behavior, social status, and sensation seeking, none which differed by group. Shown in Table 1, non-significant treatment group differences for five item self-esteem scale had small to moderate effect sizes of 0.6 in Study 1 and 0.25 in Study 2; effect sizes for the four-item susceptibility to peer pressure scale were 0.6 in Study 1 and 0.09 in study 2. These findings are consistent with successful randomization.

**Table 1:** Pre-drive check on randomization and post-drive check on passenger social norms.

		Excluded + passive passenger			Excluded + risk-accepting passenger				Included (fair play + risk-accepting passenger				
		*N*	Mean	*SD*	*N*	Mean	*SD*	Effect size §	*N*	Mean	*SD*	*p*	Effect size §§
	**Measure (items; range)**	**Pre-drive randomization check^†^**											

Study 1: males (*N* = 112)	Self-esteem^ns^ (1; 1–5)	40	4.03	0.83	36	4.28	0.61	0.34	36	3.94	0.53	0.10	0.60
	Susceptibility to peer pressure^ns^ (10; 1–4)	40	1.69	0.52	36	1.85	0.41	0.34	36	1.81	0.53	0.33	0.63
		**Post-drive check on norms manipulation/perceptions of confederate passenger^‡^**											
	Identification with passenger^∗∗∗^ (6; 1–3)	39	1.99	0.40	36	2.39^aaa^	0.47	0.92	36	2.30^a^	0.50	<0.001	0.19
	Passenger approval of risky driving^∗∗^ (5; 1–5)	39	2.83	0.68	36	4.13^aaa^	0.73	1.84	36	4.25^aaa^	0.70	<0.001	0.17

		**Pre-drive randomization check^†^**											

Study 2: females (*N* = 115)	Self-esteem^ns^ (1; 1–5)	39	3.63	0.71	39	3.62	0.94	0.01	37	3.38	0.98	0.39	0.25
	Susceptibility to peer pressure^ns^ (10; 1–4)	39	1.59	0.45	39	1.73	0.46	0.31	37	1.77	0.47	0.23	0.09
		**Post-drive check on norms manipulation/perceptions of confederate passenger^‡^**											
	Identification with passenger^∗∗∗^ (6; 1–3)	39	2.04	0.26	39	2.27	0.47	0.61	37	2.46	0.34	<0.001	0.46
	Passenger approval of risky driving^∗∗^ (5; 1–5)	39	2.47	0.50	39	4.12	0.84	2.39	37	4.47	0.50	<0.001	0.51

*^†^Prior to eventual group assignment; ^‡^Administered after randomization and experimental drives. One-way ANOVA; *post hoc* comparisons with Tukey–Kramer adjustment; ^*ns*^*p* > 0.05, ^∗∗^*p* < 0.01, ^∗∗∗^*p* < 0.001 for overall model. ^*a*^*p* < 0.05, ^*aaa*^*p* < 0.001 when compared to excluded + passive passenger group. § Standardize mean differences for Excluded + passive passenger vs. Excluded plus risk-accepting passenger; §§ Standardized mean differences for Excluded plus risk-accepting vs. Included vs. risk-accepting passenger.*

### Randomization and Confederate Passenger Manipulation

The top half of Table 1 for Study 1 (males) and bottom half for Study 2 (females) show the post-treatment values for identification with the passenger and perceived passenger approval of risky driving, assessed the success of the confederate passenger manipulation. Participants in both studies were more likely to identify with the risk-accepting passenger than the passive passenger, with effect sizes for the scale with response options of 1–3 of 0.47 (moderate) and 0.19 (small) in Study 1 and 0.47 (moderate) and 0.46 (moderate) in Study 2, consistent with previous research (Simons-Morton et al., 2014). Also, in both studies participants perceived that the risk accepting passenger was more approving of risky driving than the passive passenger, with effect sizes on the scale with response options ranging 1–5 of 0.73 (moderate to large) and 0.17 (small) in Study 1 and 0.84 (large) and 0.51 (moderate) in Study 2, consistent with the planned manipulation of confederate passenger norms.

### Manipulation of Exclusion

Shown in Table 2 are the values for each study assessing the Cyberball manipulation and psychological needs variables immediately after Cyberball. Means in response to the question, “How much did they throw you the ball?” were higher (one-way ANOVA, three groups) for the inclusion (fair play) than the exclusion groups in both studies, with small effect sizes of 0.20 and 0.16 when the two excluded groups were compared and large effect sizes of 1.06 and 1.02 when the included group was compared to the included (fair play) group, consistent with successful manipulation of exclusion. Need threat values were somewhat higher in Study 1 (males) than in Study 2 (females), but in both studies the values did not differ between the two exclusion groups and were lower in the exclusion groups than the inclusion (fair play) group; moderate to large effect sizes of 0.44 to 1.55 across the two studies for inclusion (fair play) with risk accepting passenger vs. exclusion with risk accepting passenger, as expected and consistent with successful manipulation.

**Table 2:** Cyberball manipulation check and need threat scores after exclusion or inclusion.

						Exclusion				Inclusion (fair play)				
			Excluded + passive passenger			Excluded + risk- accepting passenger				Included (fair play) + risk-accepting passenger				
	Measures (item; range)	Alpha	*N*	Mean	*SD*	*N*	Mean	*SD*	Effect size §	*N*	Mean	*SD*	*p*	Effect size §§
Study 1: males (*N* = 112)	Cyberball manipulation check (1; 1–5)	–	36	2.87	0.80	39	2.72	0.66	0.20	36	3.50^aa,bb^	0.81	<0.001	1.06
	Belongingness(5; 1–7)	0.81	36	3.30	0.95	40	3.20	1.03	0.10	35	4.79^aaa,bbb^	1.11	<0.001	1.48
	Self-esteem (5; 1–7)	0.76	36	4.58	0.95	40	5.13	0.99	0.57	35	5.61^aaa^	1.17	<0.001	0.44
	Control (5; 1–7)	0.82	36	3.41	0.90	40	2.90	1.21	0.48	35	4.61^aaa,bbb^	1.06	<0.001	1.50
	Meaningful existence (5; 1–7)	0.86	36	4.05	1.16	40	3.85	1.26	0.17	35	4.83^a,bb^	1.30	<0.01	0.75
Study 2: females (*N* = 115)	Cyberball manipulation check (1; 1–5)	–	39	2.56	0.79	39	2.69	0.83	0.16	36	3.54^aaa, bbb^	0.84	<0.001	1.02
	Belongingness(5; 1–7)	0.87	39	2.81	1.06	38	2.81	1.18	0.00	37	4.38^aaa, bbb^	1.19	<0.001	1.32
	Self-esteem(5; 1–7)	0.80	39	4.44	1.14	38	4.47	1.36	0.02	37	5.17^aa, b^	1.14	0.015	0.56
	Control (5; 1–7)	0.85	39	2.64	0.96	38	2.57	1.05	0.07	37	4.38^aaa, bbb^	1.27	<0.001	1.55
	Meaningful existence (5; 1–7)	0.89	39	3.38	1.26	38	3.60	1.37	0.17	37	4.45^aaa, bb^	1.22	0.001	0.66

*^*a*^p < 0.05, ^*aa*^p < 0.01, ^*aaa*^p < 0.001 when compared to excluded + passive passenger group; ^*b*^*p* < 0.05, ^*bb*^*p* < 0.01, ^*bbb*^*p* < 0.001 when compared to excluded + risk-accepting passenger group. § Standardized mean differences for Excluded + passive passenger vs Excluded plus risk-accepting passenger; §§ Standardized mean differences for Excluded plus risk-accepting vs Included vs risk-accepting passenger.*

### Treatment Group Differences

Shown in Table 3 (top half for Study 1 and bottom half for Study 2) are the means (and SDs) for each measure of risk for each group for the solo and experimental passenger drives. The differences in the baseline values of the three outcome variables between the three groups were not significant in ANOVA (data not shown), providing evidence of successful random assignment and consistency in the baseline simulation drives. Note that baseline measures of risky driving were higher for Study 1 males than Study 2 females, as might be expected, particularly in passing the slowing vehicle, with (30 to 34% of males and only 3 to 11% of females passing). The last three rows for each study show the differences between the solo and the passenger drive for each measure and group, values that are useful for interpreting the treatment group differences. Note declines from baseline to passenger drive for excluded participants, at least for the two intersection tasks, and increases for most measures among included participants.

**Table 3:** Mean values for each drive and measure of risk (unadjusted).

	Measure	Excluded + passive passenger			Excluded + risk-accepting passenger			Included (fair play) + risk-accepting passenger		
		***N***	**Mean**	***SD***	***N***	**Mean**	***SD***	***N***	**Mean**	***SD***
	
Study 1: males (*N* = 112)	Failure to stop – baseline solo (%)	40	59.25	40.91	36	48.61	36.27	36	55.00	37.83
	Failure to stop – experiment/passenger (%)	40	50.00	43.85	36	46.39	39.51	36	58.06	37.33
	Percent time in red – baseline solo (%)	40	36.30	25.67	36	28.92	22.22	36	32.73	24.18
	Percent time in red – experiment/passenger (%)	40	28.75	26.25	36	25.49	22.59	36	32.63	22.58
	Passed slow vehicle – baseline solo (%)	40	30.00	46.41	35	34.29	48.16	35	34.29	48.16
	Passed slow vehicle – experiment/passenger (%)	40	27.50	45.22	36	52.78	50.63	36	52.78	50.63
	Difference failed to stop	40	–9.25	19.13	36	–2.22	20.02	36	3.06	16.87
	Difference percent time in red	40	–7.56	12.63	36	–3.44	11.77	36	–0.10	11.21
	Difference pass slow vehicle	40	–0.03	0.36	35	0.17	0.45	35	0.17	0.51
Study 2: females (*N* = 115)	Failure to stop – baseline solo (%)	39	46.41	37.10	39	47.69	35.05	37	55.94	31.57
	Failure to stop – experiment/passenger (%)	39	38.97	34.24	39	44.87	35.16	37	59.46	33.50
	Percent time in red – baseline solo (%)	39	27.48	22.73	39	29.25	22.16	37	33.61	20.83
	Percent time in red – experiment/passenger (%)	39	21.14	19.78	39	25.39	20.42	37	34.11	20.43
	Passed slow vehicle – baseline solo (%)	39	2.56	16.01	39	10.26	30.74	37	10.81	31.48
	Passed slow vehicle – experiment/passenger (%)	39	2.56	16.01	39	20.51	40.91	37	16.22	37.39
	Difference failed to stop	39	–7.44	24.03	39	–2.82	21.14	37	3.51	18.14
	Difference percent time in red	39	–6.34	15.19	39	–3.86	14.04	37	0.54	11.21
	Difference pass slow vehicle	39	0.0	0.0	39	10.26	30.74	37	5.41	32.88

### Effect of Passenger Type

In Table 4 the columns on the left show the estimates for the effect of passenger type on the three risky driving measures among participants in the two exclusion groups, adjusted for self-esteem. In Study 1 (males) there were significant effects of passenger type for 2 of 3 measures, % Not Stopping for Red Lights (OR = 2.09), which declined from 59.3% at baseline to 50.0% in the passive passenger group and from 48.6 to 46.4% for the risk accepting passenger group (see Table 3), and % passing the slowing vehicle (OR = 3.41), which declined from 30.0 to 27.5% in the passive passenger group and increased from 34.3 to 52.8% in the risky passenger group, consistent with an effect of increased risk in the presence of the risk accepting passenger. The effect sizes were large for all three measures of risky driving.

**Table 4:** Treatment group differences^∗^.

		Excluded + risk-accepting passenger vs. Excluded + passive passenger (effect of risk-accepting passenger)				Excluded + risk-accepting passenger vs. Included (fair play) + risk-accepting passenger (effect of exclusion)			
		**Est.**	**95% CI**		***p*-value**	**Est.**	**95% CI**		***p*-value**
	
Study 1: males (*N* = 112)	Failed to stop (OR)	2.09	1.14	3.81	**0.02**	0.63	0.35	1.12	0.11
	Percent time in red (β)	3.60	–0.72	7.93	0.10	–3.73	–8.22	0.75	0.10
	Pass slow vehicle (OR)	3.41	1.03	11.35	**0.05**	1.04	0.32	3.42	0.94
Study 2: females (*N* = 115)	Failed to stop (OR)	1.40	0.84	2.32	0.19	0.60	0.36	0.98	**0.04**
	Percent time in red (β)	2.56	–2.24	7.36	0.30	–5.30	–10.16	–0.45	**0.03**
	Pass slow vehicle (OR)	9.94	1.16	85.31	**0.04**	1.46	0.44	4.81	0.53

*^∗^Controlling for self-esteem; Odds ratio is the effect size for GLIMMIX model with binary outcomes and the beta is the effect size for mixed models. The significant (<0.05) p values are in bold.*

In Study 2 female drivers were significantly more likely to pass the slowing vehicle in the presence of a risk accepting passenger, increasing from 10 to 21%, but not changing among those driving with the passive passenger (see Table 3). Overall, the treatment group comparisons for the risky driving variables favored increased risky driving among those exposed to the risk accepting passenger on 2 of 3 measures for males and 1 of 3 for females, with large effect sizes.

### Effect of Exclusion vs. Inclusion (Fair Play)

The right half of Table 4 show the treatment group differences for each risky driving measure for participants in the excluded and included (fair play) groups in the presence of a risk accepting passenger. In Study 1 (males) no significant treatment group differences were found, although % Not Stopping and % Time in Red (*p* = 0.11 and *p* = 0.10) had moderate effect sizes favoring increased risk in the inclusion (fair play) group, with slight declines in the exclusion group and slight increases or little change in the inclusion (fair play) group. Analyses adjusted for susceptibility to peer pressure resulted in negligible differences in the estimates (available upon request).

For females in Study 2, shown in the right half of Table 4, % Not Stopping (*B* = 0.60, *p* = 0.04) was significant, with declines in the socially excluded group (from 47.7 to 44.9%) and increases in the socially included (fair play) group (from 55.9 to 59.4%), and % Time in Red was significant (*B* = -5.30, *p* = 0.03) with a decline from 29.3 to 25.4% in the excluded group and an increase from 33.6 to 34.1% in the socially included group.

## Discussion

This research examined influences of peer passengers and social exclusion on simulated risky driving among male and female teenagers. Identical trials were conducted separately with males and females, in which participants were randomized to one of three treatment conditions allowing evaluation of the following research questions about simulated risky driving: (a) What is the effect of exposure to a risk accepting or passive passenger after social exclusion? (b) What is the effect of social exclusion or social inclusion (fair play) when exposed to a risk accepting passenger? We discuss the findings for each trial (males and females) in relation to each research question.

### Peer Influence on Risky Driving After Social Exclusion

To test the possible effect of peer influence on risky driving, it was necessary for participants to perceive differences in the risk acceptance of the confederate passengers. In post-treatment analyses, both males and females identified more strongly with the risk accepting passenger and perceived that the risk accepting passenger was more approving of risky driving than the passive passenger, consistent with successful manipulation of confederate passenger norms, thus allowing for logical interpretation of passenger effects. Accordingly, the findings generally support increased simulated risky driving in the presence of a risk accepting passenger after social exclusion. For males, 2 of the 3 risky driving variables, not stopping for the red light and passing the slowing vehicle, indicated significantly greater risk among those exposed to a risk accepting passenger relative to those exposed to a risk passive passenger. For females, 1 of 3 measures, passing the slowing vehicle, indicated significantly greater risk among those exposed to a risk accepting passenger, consistent with conformity to social norms. Hence, 2 of 3 variables for males and 1 of 3 for females indicated greater risky driving in the presence of a risk accepting passenger relative to a passive passenger after social exclusion, with moderate or large effect sizes.

These findings are generally consistent with social norms theory (Simons-Morton et al., 2009), with participants conforming in their driving behavior to passenger norms regarding risky driving. These findings are also consistent with previous simulation trials that found that simulated risky driving was greater among young males in the presence of risk accepting confederate peers who exerted mild pressure to drive in a more risky manner, which the authors attributed to peer pressure and social norms (Bingham et al., 2016); and in the presence of risk accepting confederate peers who exerted no explicit pressure during the drive, which the authors attributed to perceived social norms (Simons-Morton et al., 2014). Other research found that simulated risky driving was greater among young males and females in the presence of their own peers (Ross et al., 2016), attributable to greater reward sensitivity in the presence of peers, similar to the finding of Chein et al. (2011), who reported greater risky driving among teens (compared to adults) whose peers observed them driving a desktop simulator.

### Social Exclusion or Social Inclusion (Fair Play) in the Presence of a Risk Accepting Passenger

Both male and female participants in the social inclusion (fair play) group reported being passed the Cyberball more than those in the exclusion group, consistent with successful manipulation of exclusion. In addition, both male and female participants in the inclusion (fair play) groups reported consistently higher scores than those in the exclusion groups on need threat variables (representing lower psychological needs) consistent with previous research indicating similar post-Cyberball need threat scores for both males and females (Pharo et al., 2011; Pharo, 2012). The findings for social exclusion on driving behavior generally favored increased risk for those included than for those excluded. For male participants, there were trends in two variables, with declines in risk from baseline among those in the exclusion group and no change in the inclusion group. For female participants, there were significant differences in two measures, with declines in the exclusion group and increases or no change in the inclusion (fair play) group. These findings are counter to our expectation that exclusion (vs. inclusion) would increase risk taking in the presence of risk accepting peers because conformity is a good way to gain, regain, or increase social acceptance after social exclusion (Williams, 2007). There are several possible explanations.

Some literature suggests that teenage males exert greater peer influence than females on both teenage males and females (Jacobs et al., 2017) and females may be more susceptible to peer influences, particularly from opposite sex friends (Dick et al., 2007). Viewed from this perspective, our findings suggest that social inclusion in the form of fair play, particularly among females, might reduce inhibition and increase susceptibility to peer influence in the presence of male peers with risk accepting attitudes. This is consistent with research suggesting specific boundary conditions on the effects of exclusion, such that participants who anticipate easily connecting with others are more likely to conform (Maner et al., 2007), and presumably inclusion (fair play) would increase the anticipation of easily connecting with others. Thus, teenagers who experienced fair play may have been more confident than teenagers who were excluded about connecting with the risk accepting male passenger and this effect might have been stronger among females than males. This possibility seems particularly likely given that the differences in risk taking between excluded and not-excluded participants are driven both by increases in risk taking in the inclusion (fair play) group and decreases in the excluded group.

Alternatively, other research has shown that being rejected does not always cause affiliative behaviors, but instead can cause antisocial responses, not only toward the excluder but also toward neutral others (Twenge et al., 2001). A large body of research demonstrates that feelings of arousal or threat can carry across situations, encouraging the exertion of control over non-social sources of threat. For example, chronic rejection is associated with decreases in school engagement among school age children (Buhs et al., 2006). Thus, it is possible that our exclusion priming threatened participants’ sense of safety and well-being, causing them to retreat and conform less to the social norms of the risk accepting confederate passenger by driving more cautiously. Relatedly, Park and Baumeister (2015) reported an increase in cautious response bias with social exclusion among adults. Their recognition task was designed to identify a preference for finding correct answers (at the risk of including some incorrect responses) or a preference for avoiding mistakes. The excluded group sought to avoid mistakes (cautious response bias) and hesitated for longer before responding whereas the included group favored finding correct answers (risky response bias).

Study strengths include experimental design using a high-fidelity simulator and proven risky driving protocol applied in separate studies with males and females. Moreover, our experimental manipulations were successful in that participants who were excluded reported lower values on the need threat measures; participants perceived the risk accepting passenger to be more accepting of risk than the passive passenger; and participants identified post-treatment with the risk accepting confederate passenger relative to the passive passenger.

The primary study limitation is the lack of a full factorial design (due to budget and time limitations), which would have provided a more elegant and complete test of passenger (with male and female participants exposed to male and female peers) and exclusion vs. inclusion (fair play) effects. We did not actually manipulate inclusion by allowing the participants who experienced fair play that others were being excluded, so our inclusion condition was actually a fair play or not-exclused condition. Also, the protocol called for the passive passenger to be neutral with respect to risk, but it is possible the participants interpreted passiveness as rejection, which could have affected their behavior, although we found no evidence of this possibility.

## Conclusion

After being socially excluded, male and female teenage study participants engaged in relatively greater risky simulated driving in the presence of a risk accepting compared to a passive passenger, consistent with social norms theory and previous research. Teenage female study participants in the presence of a risk-accepting passenger engaged in more risky driving after experiencing fair play, compared to those who had been socially excluded, contrary to prevailing theory; males exhibited similar but non-significant trends. These findings provide additional support for the contention that social norms influence teenage risky driving behavior, indicate that inclusion might increase and exclusion might reduce risk taking behavior in the presence of a risk-accepting male peer, suggesting that social relationships among teens matter with respect to their influence on risk behavior. The findings suggest important new avenues for research on gender differences with respect to the effects of social exclusion on adolescent risk behavior.

## Ethics Statement

The study was reviewed and approved by the University of Michigan IRB. Written and informed consent was obtained from the parents of all participants and assent was obtained from all participants.

## Author Contributions

BS-M, CB, and JS conceptualized the study, obtain funding, and participated in the conduct, data analyses, and manuscript preparation. KL and CZ participated in methods development, analyses, and manuscript preparation. LB and EF participated in analyses and manuscript preparation.

## Conflict of Interest Statement

The authors declare that the research was conducted in the absence of any commercial or financial relationships that could be construed as a potential conflict of interest.
