# Supplementary figures and images for: The Effect of Teenage Passengers on Simulated Risky Driving Among Teenagers: A Randomized Trial
Source: Front Psychol. 2019 Apr 30;10:923. doi: 10.3389/fpsyg.2019.00923 (PMC6524721; doi:10.3389/fpsyg.2019.00923)

Appendix 1. Driving simulator cockpit as driver approaches a signalized intersection.


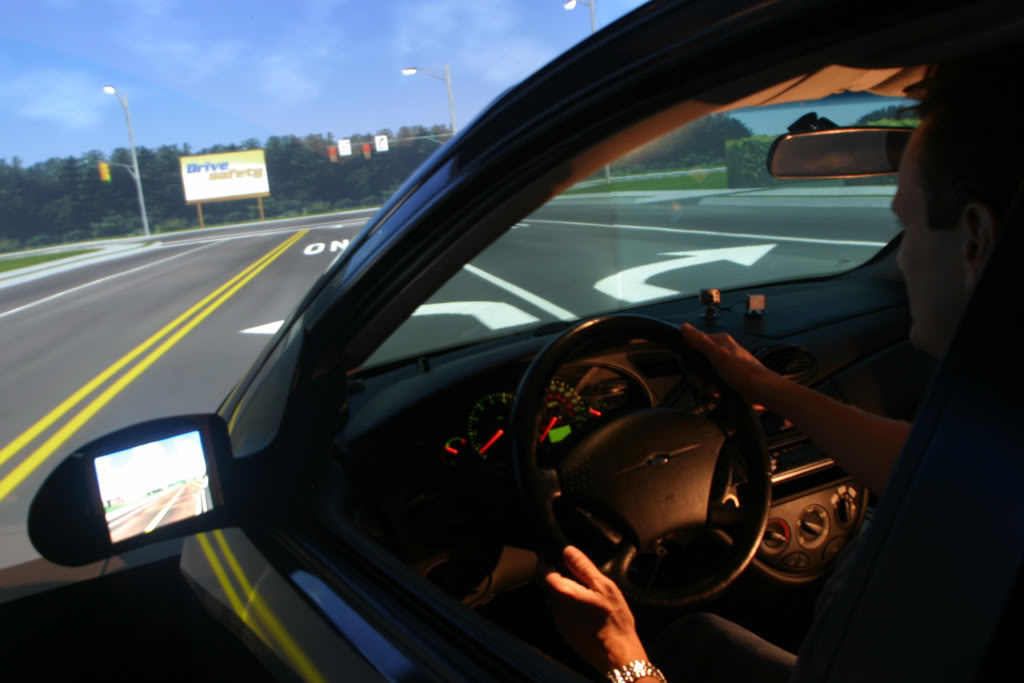

Supplement: Supplementary file 1 [file Table_1.DOCX]
